# The anti-inflammatory effect of bacterial short chain fatty acids is partially mediated by endocannabinoids

**DOI:** 10.1080/19490976.2021.1997559

**Published:** 2021-11-17

**Authors:** Amrita Vijay, Afroditi Kouraki, Sameer Gohir, James Turnbull, Anthony Kelly, Vicky Chapman, David A Barrett, William J Bulsiewicz, Ana M Valdes

**Affiliations:** a Division of Rheumatology, Orthopedics and Dermatology, School of Medicine, University of Nottingham, Nottingham, UK; b Nihr Nottingham Biomedical Research Centre, University of Nottingham, Nottingham, UK; c Arthritis Research Uk Pain Centre, University of Nottingham, Medical School, Queen’s Medical Centre, Nottingham, UK; d DAB-Centre for Analytical Bioscience, Advanced Materials and Healthcare Technologies Division, School of Pharmacy, University of Nottingham, Nottingham, UK; e Lowcountry Gastroenterology Associates, Mount Pleasant, South Caroline, USA

**Keywords:** Endocannabinoids, exercise, gut microbes, SCFA, inflammatory markers, intervention

## Abstract

The endocannabinoid (EC) system has pleiotropic functions in the body. It plays a key role in energy homeostasis and the development of metabolic disorders being a mediator in the relationship between the gut microbiota and host metabolism. In the current study we explore the functional interactions between the endocannabinoid system and the gut microbiome in modulating inflammatory markers. Using data from a 6 week exercise intervention (treatment n = 38 control n = 40) and a cross sectional validation cohort (n = 35), we measured the associations of 2-arachidonoylglycerol (2-AG), anandamide (AEA), *N*-oleoylethanolamine (OEA) and *N*-palmitoylethanolamine (PEA) with gut microbiome composition, gut derived metabolites (SCFAs) and inflammatory markers both cross-sectionally and longitudinally. At baseline AEA and OEA were positively associated with alpha diversity (β(SE) = .32 (.06), *P* = .002; .44 (.04), *P* < .001) and with SCFA producing bacteria such as *Bifidobacterium* (2-AG β(SE) = .21 (.10), *P* < .01; PEA β(SE) = .23 (.08), *P* < .01), *Coprococcus 3* and *Faecalibacterium* (PEA β(SE) = .29 (.11), *P* = .01; .25 (.09), *P* < .01) and negatively associated with *Collinsella* (AEA β(SE) = −.31 (.12), *P* = .004). Additionally, we found AEA to be positively associated with SCFA Butyrate (β(SE) = .34 (.15), *P* = .01). AEA, OEA and PEA all increased significantly with the exercise intervention but remained constant in the control group. Changes in AEA correlated with SCFA butyrate and increases in AEA and PEA correlated with decreases in TNF-ɑ and IL-6 statistically mediating one third of the effect of SCFAs on these cytokines. Our data show that the anti-inflammatory effects of SCFAs are partly mediated by the EC system suggesting that there may be other pathways involved in the modulation of the immune system via the gut microbiome.

## Introduction

Exercise is known to elicit a feeling of euphoria, referred to as a “runner’s high”, which recent studies indicate is the result of activation of the endocannabinoid system.^1,2^ Endocannabinoids (ECs), such as anandamide (AEA), 2-arachidonoylglycerol (2-AG), N-palmitoylethanolamine (PEA), and N-oleoylethanolamine (OEA) are lipid mediators that bind to specific receptors and elicit cell signaling. The EC system modulates systemic energy metabolism, inflammation, pain, and brain biology^3^ and is comprised of ECs, its receptors, most notably the G-protein coupled receptors CB1, CB2, and the enzymes that produce and degrade ECs.^4^ The role of this system in modulating inflammation,^5^ muscle strength^6,7^ and energy metabolism^8,9^ is now widely documented in humans and in other mammals.^10^

In addition, there is a vast body of evidence suggesting that the gut microbiome and exercise are interconnected to regulate metabolism and homeostasis, independent of diet.^11^ Specifically, exercise has been shown in both animal model and human studies to increase the relative abundance of butyrate-producing microbes and thereby increase the production of butyrate, a short chain fatty acid with systemic anti-inflammatory benefits.^12–17^

Separately, the gut microbiome and the EC system have also been connected to metabolic regulation and homeostasis.^18^ For over a decade now it has been known that specific gut microbial strains modulate the expression of cannabinoid and μ-opioid receptors in intestinal cells.^19^ Extensive work in animal models has shown that gut microbes also counteract obesity-induced overactivity of the EC system in the mouse colon, with subsequent reduction of gut permeability to lipopolysaccharide

(LPS) (i.e. decreased metabolic endotoxaemia) and increased adipogenesis.^20,21^ Accordingly, prebiotics, probiotics and antibiotics affect the intestinal EC system. These effects of the microbiota appear to be mediated in part by the modulation of EC inactivating enzymes, which also metabolize EC-related mediators with activity at non-cannabinoid receptors.^22^ Furthermore, dysregulation of the EC system has been connected to digestive disorders such as inflammatory bowel disease, irritable bowel syndrome, as well as obesity.^23–25^ These conditions involve both a dysregulated microbiota (dysbiosis) and altered short chain fatty acids (SCFAs) levels.^26–28^

To date the mechanisms and the extent to which the anti-inflammatory effects of gut microbial production of (SCFAs) are mediated or induced by changes in the EC system have not been explored. Moreover, the links between changes in specific bacterial strains and EC levels in response to dietary or other interventions are lacking in humans. In this study we have investigated the cross-sectional links between ECs and gut microbiome composition in two cohorts and further investigated the relationship between changes in ECs and gut microbiome in response to an exercise intervention.

## Methods

### Study population

The longitudinal cohort belonged to community dwelling individuals (Age: >45 y) as part of the iBEATOA study.^29^ The cross-sectional cohort was an independent cohort consisting of healthy individuals aged >18 y. The associations of ECs with microbiome and metabolomic data was carried out cross-sectionally first in the longitudinal cohort and then validated in the independent cohort consisting of healthy volunteers. Longitudinal analysis was performed using baseline and follow-up data in the webex cohort.^29^

All participants provided written informed consent. For the longitudinal cohort, ethical approval was obtained from the Research Ethics Committee (ref:18/EM/0154) and the Health Research Authority (protocol no: 18021) and the trial is registered under the clinicaltrials.gov database (NCT03545048). For the cross-sectional (validation cohort), ethical approval was sought by the West Midlands Black Country Research Ethics Committee (18/WM/0066).

### Sample collection

Baseline blood, stool, and anthropometric measures (such as, height and weight) were collected in both cohort studies. Blood samples were collected from participants between 8:30am and 10am during each visit. Participants were instructed to come in a fasted state at least since 9 pm the night before (i.e. minimum fasting time was 11.5 hours). Blood samples were collected using Serum Separator Tubes (SST) and were processed within 2–3 hours of collection for separating serum and aliquoted for storage at −80 C until the end of the intervention period.

### Metabolomic analysis

#### Lipidomic measurements

2-AG, AEA, PEA and OEA measurements were extracted from .5 ml serum samples and quantified against a fully extracted calibration line using targeted liquid chromatography tandem mass spectrometry (LC-MS/MS) based on the method described previously.^30^

#### Serum short-chain fatty acids

Serum SCFAs in the Webex study cohort were measured using the standardized procedures by Metabolon Inc., Durham, USA.^31^ Serum SCFA in the validation study cohort were measured by the Mass Spectrometry Department, King’s College London using in-situ pentafluorobenzylation of the  free acid species, followed by gas chromatography-negative-chemical-ionization mass spectrometry (GC-NCI-MS) determination of the resulting derivatives as described previously.^32,33^

#### Inflammatory markers

Pro and anti-inflammatory serum markers were measured by Affinity Biomarkers, London using the standardized Human Proinflammatory panel 1 assay kit (cat number K151A0H-1), distributed by Meso Scale Discovery as described previously.^33^

### Gut microbiome sequencing

Fecal sample collection and processing methods were the same in both cohort studies. Fecal samples were collected by the participant at home using previously provided collection kits and frozen immediately at −80°C until further processing. Stool DNA extraction was carried out according to Goodrich et al.^34^ using100 mg of the stool sample. There was no homogenization prior to this step. Gut microbiome composition was determined by 16 S rRNA gene sequencing carried out as previously described.^32,33^ Briefly, the V4 region of the 16S rRNA gene was amplified using universal primers 355 F (CCAGACTCCTACGGGAGGCAGC) and 806 R (GGACTACHVGGGTWTCTAAT). Amplified DNA was sequenced on the MiSeq platform (Illumina, 300bp paired-end reads). Read filtering and clustering were carried out using the MYcrobiota pipeline. Briefly, chimeric sequences were filtered using the VSEARCH algorithm within Mothur, and reads were clustered into operational taxonomic units (OTUs) using closed-reference clustering against the SILVA database v132 based on a 97% similarity. Diversity metrics (Shannon index observed OTUs and Unweighted UniFrac) were calculated by rarefying the OTU table down to 7000 sequences per sample 50 times and taking the average. These analyses were carried out in QIIME2 (v2018.11).

### Gene expression

Barcoded libraries for RNA-seq were prepared with 5ng of RNA using TruSeq Stranded Total RNA HT Sample Prep Kit with Ribo-Zero Gold kit (Illumina) per manufacturer’s protocol. Paired-end sequencing (100 bp × 2) was performed on HiSeq 4000 sequencers (Illumina) at Genewiz (UK).^35^

### Statistical analysis

All statistical analyses were carried out in R v4.0.3. OTUs with a relative abundance of <.1% in every sample were removed, and zero inflated relative OTU abundances were inverse normal transformed before further analyses. All analyses were adjusted for age, gender and BMI and multiple testing using false discovery rate (FDR q < .05). Linear regressions were first carried out independently in both cohorts to identify significant associations between endocannabinoids, gut microbiota composition, SCFAs and cytokines along with adjusting for covariates such as age, sex, BMI and multiple testing (FDR q < .05). The standardized estimates were meta-analyzed to produce a combined effect after adjusting for covariates and multiple testing. Meta-analysis takes the effect size, standard error and sample size into account to give an overall effect from the different groups studied. For the cross-sectional analysis, we used fixed-effects inverse-variance models since our cohorts were homogenous having adjusted for age, sex and BMI.

## Results

The descriptive characteristics of the cohorts are shown in Table 1.

**Table 1.** 

	Longitudinal cohort (Webex) (N = 78)				Cross-sectional cohort (N = 35)	
	Control group (n = 40)		Exercise group (n = 38)			
	Baseline Mean (± SD)	Follow-up Mean (± SD)	Baseline Mean (± SD)	Follow-up Mean (± SD)	Mean (± SD)	
*Demographics*						
Age (y)	67.59 (9.71)		65.32 (10.08)		69.51 (8.77)	
Men/Women (%)	8/32 (20/80)		10/28 (26/73)		10/25 (32/68)	
BMI (kg/m^2^)	32.86 (7.80)		32.58 (7.50)		29.81 (5.12)	30.31 (7.26)
*Endocannabinoids*						
2-AG	52.40 (42.12)	49.32 (32.13)	42.50 (28.85)	44.61 (30.48)	41.91 (36.81)	
AEA	1.68 (.38)	1.53 (.26)	1.61 (.35)	1.79 (.44)**	.94 (.34)	
OEA	7.18 (1.49)	7.15 (2.03)	6.71 (1.87)	7.37 (2.13)**	4.48 (1.78)	
PEA	21.99 (33.22)	19.19 (16.99)	26.41 (24.14)	42.07 (31.18)**	26.03 (21.92)	
*Cytokines*						
INFγ	18.46 (17.91)	17.15 (15.63)	14.29 (10.80)	11.25 (7.30)	12.71 (8.12)	
IL-10	1.02 (1.69)	.79 (.76)	.55 (.36)	.42 (.31) *	.67 (.53)	
IL-13	3.44 (2.49)	2.91 (1.15)	3.26 (1.54)	1.81 (.98)**	3.18 (2.71)	
IL-1	.34 (.45)	.22 (.15)	.28 (.27)	.18 (.09)	.41 (.39)	
IL-4	.34 (.62)	.32 (.21)	.30 (.57)	.10 (.03)**	.15 (.09)	
IL-6	3.16 (2.93)	2.82 (2.74)	2.67 (.86)	2.02 (.71)	1.42 (1.22)	
IL-8	41.75 (35.62)	40.04 (35.84)	42.50 (2.21)	32.80 (2.20)	31.79 (17.19)	
TNFα	7.05 (6.46)	6.44 (6.03)	7.78 (9.01)	5.91 (3.52)*	3.37 (.89)	
*SCFAs*						
Acetic acid	28.98 (17.89)	26.92 (16.45)	33.61 (18.43)	*32.38 (17.12)*	43.43 (68.69)	
Butyric acid	11.04 (2.53)	10.63 (2.74)	*10.86 (2.67)*	17.23 (4.10)*	8.39 (.73)	
Propionic acid	2.28 (5.98)	2.28 (6.17)	*2.42 (6.57)*	6.91 (2.03)*	10.13 (.79)	
Valeric acid	3.21 (7.33)	2.92 (7.7)	*3.20 (4.31)*	*3.38 (4.63)*	1.25 (.52)	
Iso-butyric acid	10.39 (2.90)	10.65 (3.09)	*9.67 (2.35)*	11.79 (3.47)*	10.69 (5.70)	
Iso-valeric acid	5.69 (3.17)	5.95 (3.02)	*5.55 (2.52)*	*5.61 (2.30)*	7.26 (2.45)	

BMI (Body Mass Index); SCFA (Short Chain Fatty Acids)

*p < .05; **p < .001. *p* values are FDR corrected obtained from paired matched t test between baseline and follow-up.

**Figure 1.**
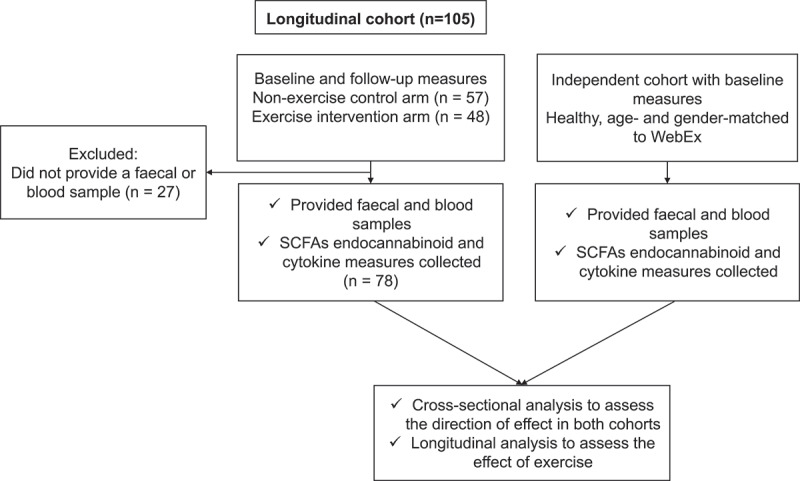
CONSORT flow diagram

The associations of endocannabinoids with gut microbiome composition, SCFAs and inflammatory cytokines were assessed both cross-sectionally and longitudinally using two independent cohorts that were matched for age and gender as described in Figure 1.

### Cross-sectional and reproducible associations of endocannabinoids with gut microbiome composition, SCFAs and inflammatory markers

**Figure 2.**
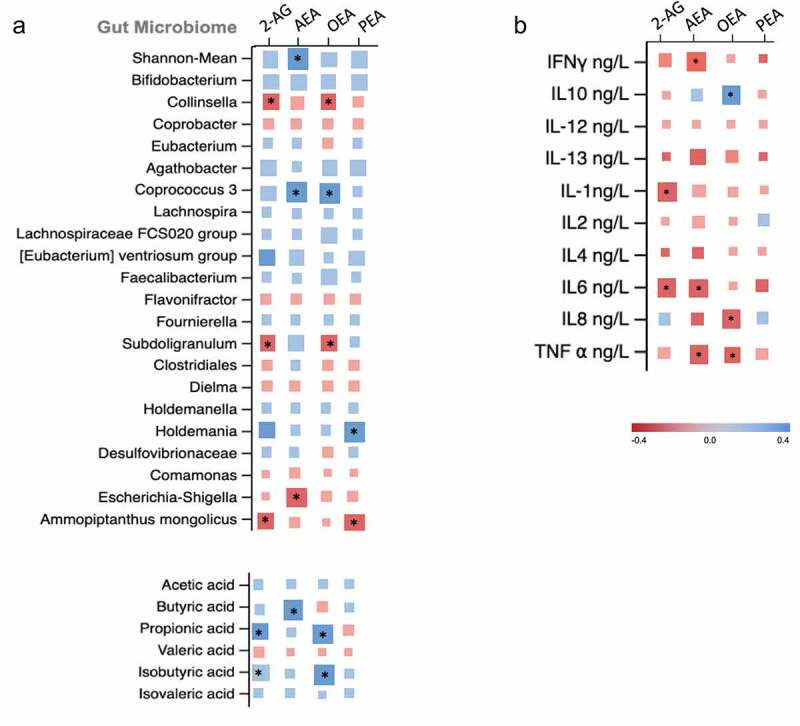
Matrix plot showing the associations of: (a) OTU abundance and short chain fatty acids with endocannabinoids; and (b) pro and anti-inflammatory markers with endocannabinoids. Associations are based on data from the longitudinal cohort. Squares represent beta coefficients with size and color varying based on size and direction of association. (FDR adjusted *p < .05)

**Figure 3.**
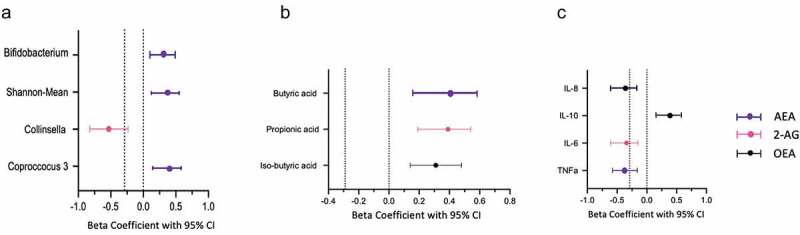
Meta-analysis of beta coefficients of with 95% CIs from cross-sectional analysis derived from both cohorts. The figure represents the strongest associations of ECs with (a) Microbiome composition; (b) Short chain fatty acids and (c) Pro and anti-inflammatory cytokines

We first carried a cross-sectional analysis to investigate the associations of endocannabinoid levels with gut microbiome composition, short chain fatty acids and inflammatory markers. We first tested this using the baseline data from the longitudinal cohort wherein we found all four endocannabinoids were positively associated with Shannon diversity, SCFA producing bacteria such as *Bifidobacteria* and *Coprococcus 3* and negatively associated with *Collinsella* and *Escherichia Shigella*, a pathogenic bacterium (Figure 2). We found the endocannabinoids mainly AEA, 2AG and the endocannabinoid like compound OEA to be significantly associated with butyrate (β(SE) = .38 (.10), *P* = .01), propionate (β(SE) = .31 (.08) *P* = .01) and iso-butyrate (β(SE) = .34 (.08), *P* = .02), respectively. Furthermore, EC levels were positively associated with anti-inflammatory markers such as IL-10 but negatively associated with a range of pro-inflammatory cytokines as shown in Figure 2. These findings were then validated in an independent cohort consisting of healthy individuals matched for age and gender wherein we found similar significant associations (**supplementary figure S1**). We tested reproducibility of the significant associations of ECs with the above mentioned traits by carrying out a meta-analysis by combining the direction of effects observed in both independent cohorts and found that the associations of endocannabinoids with Shannon diversity, specific OTUs, SCFAs and pro and anti- inflammatory markers were significantly reproducible as shown in Figure 3.

### Proportional variance explained by the ECs on the associations of the gut microbiome with inflammatory markers

**Figure 4.**
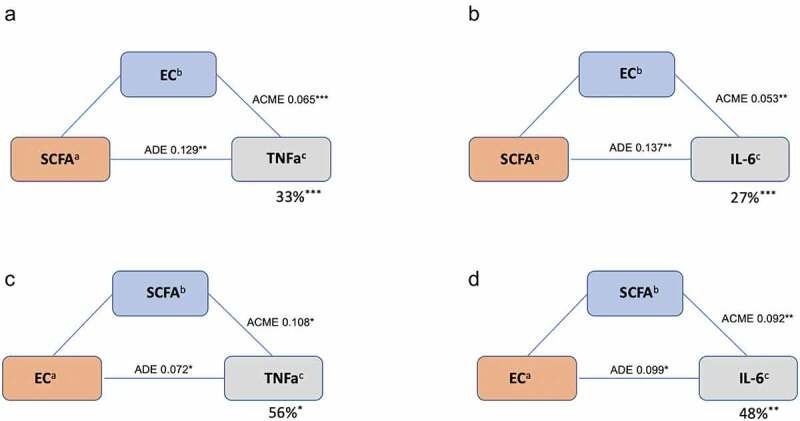
Mediatory effects of ECs and SCFAs on pro-inflammatory markers: TNFα and IL-6. (a) SCFA: butyrate, iso-butyrate (b) EC: AEA, PEA

We then explored the proportional effect of the gut microbiota and ECs on inflammatory markers that were significantly associated (FDR *p* < .05) with both these parameters. We explored these effects by formal mediation where ECs were fitted as mediator of the effect of SCFAs on inflammatory markers. Overall, we found that ECs partially mediated the association between SCFAs and inflammatory markers. AEA mediated 33% (*P* < .001) of the effect of SCFA (butyrate) on TNF〈 and 27% (*P* = .001) of the effect of SCFA on IL-6. We also tested how much of the effect of ECs on inflammatory markers is mediated by SCFA and found that 56% (*P* = .02) and 48% (*P* = .001) of SCFA mediated the effects on TNF〈 AND IL-6 respectively, as shown in Figure 4.

### Longitudinal association of endocannabinoids with gut microbiome composition, SCFAs and inflammatory markers and response to exercise

**Figure 5.**
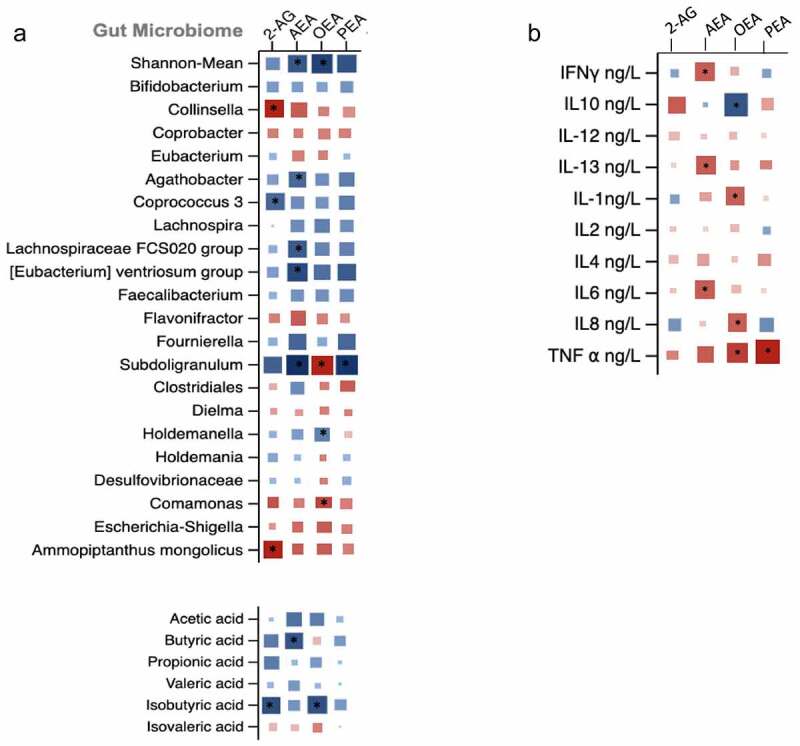
Matrix plot showing the associations of (a) change in OTU abundance and short chain fatty acids with change endocannabinoids; (b) change in pro- and anti-inflammatory markers with change endocannabinoids. Squares represent beta coefficients with size and color varying based on size and direction of association. (FDR adjusted *p < .05)

We found that levels of AEA, OEA and PEA were all significantly increased in the exercise group but not in the control group. Similarly, we observed significant decreases in proinflammatory cytokines and increases in specific SCFAs in the exercise but not in the control group (Table 1). We then assessed the correlations between changes in ECs with change in microbiome composition, SCFAs and inflammatory cytokines. The associations in the intervention arm alone did not show changes in ECs to be associated with changes in SCFAs (Supplementary Figure S2) after FDR correction. Therefore, we looked at changes from baseline to follow up using data from both arms looking at the overall change effect of ECs with SCFAs and other markers. We found that amongst the ECs, changes in AEA and OEA were positively associated with gut microbiome diversity (β(SE) = .32 (.06), *P* = .002; .44 (.04), *P* < .001). Increases in ECs were also associated with increased abundance of SCFA producing bacteria such as Bifidobacterium (2-AG β(SE) = .21 (.10), *P* < .01; PEA β(SE) = .23 (.08), *P* < .01), Coprococcus 3 and Faecalibacterium (PEA β(SE) = .29 (.11), *P* = .01; .25 (.09), *P* < .01) and negatively associated with Collinsella (AEA β(SE) = −.31 (.12), *P* = .004). Additionally, we found AEA to be positively associated with SCFA Butyrate (β(SE) = .34 (.15), *P* = .01). When we associated ECs with cytokines, we found positive associations of 2AG and OEA with anti-inflammatory markers such as IL-10 and negative associations of most ECs with some of the pro-inflammatory cytokines, TNFα and IL-6 (Figure 5).

### Association of endocannabinoids with gene expression levels

**Figure 6.**
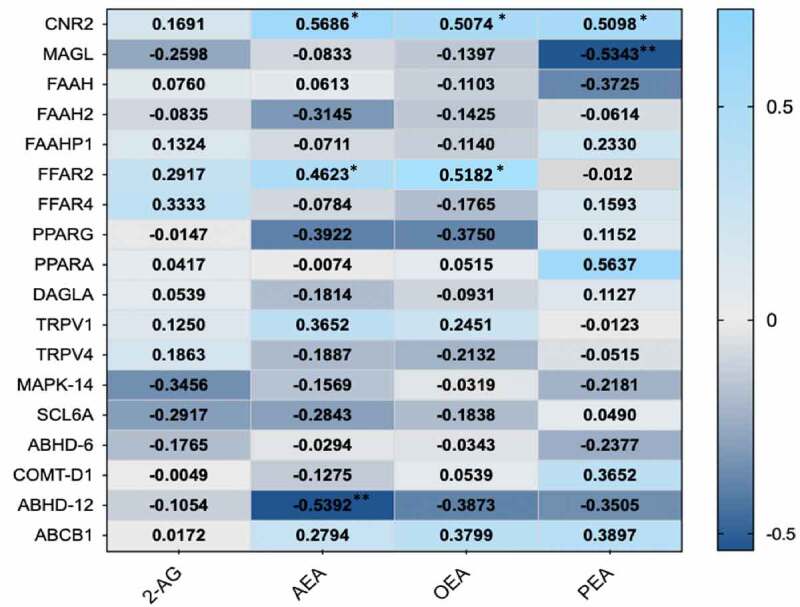
Heat map indication positive (light blue) and negative (dark blue) associations of ECs with gene expression levels of specific EC and SCFA receptor proteins. (*p < .05)

The association of endocannabinoids with gene expression levels of specific cannabinoid receptors as well as SCFA receptors from the longitudinal cohort was tested. We found most ECs to be positively associated with one of the main cannabinoid receptors CNR2 (all *P* < .05). Interestingly, AEA and OEA were found to be positively associated with FFAR2 which is one of the main SCFA fatty acid receptors (Figure 6).

## Discussion

In this study we find that approximately one third of the anti-inflammatory downstream effects of SCFAs are statistically mediated by endocannabinoids but approximately two thirds of the effect of SCFAs on cytokines appears not to be related to ECs. Using an exercise intervention, we report that increases in SCFA-producing bacteria and decreases in the pro-inflammatory genus *Collinsella* are correlated with increases in endocannabinoid circulating levels.

Previous research has shown increased abundance of *Collinsella* to be strongly associated with increased intake of processed food and low vegetable intake (Menni et al., 2021; Wolf et al., 2019), whereas decreased abundance of *Collinsella* was observed following a Mediterranean diet intervention (Ghosh et al., 2020). Furthermore, it was found that this genus significantly increased the risk of nonalcoholic steatohepatitis, the most serious form of nonalcoholic fatty liver disease whereby inflammation causes liver damage that can progress to cirrhosis (Astbury et al., 2020). Moreover, increased levels of *Collinsella* have been detected in individuals with type 2 diabetes^36^ and were noted to decrease in a weight loss study where insulin sensitivity improved during the course of the study.^37^ *Collinsella* was also positively correlated with heightened inflammatory cell count profiles (i.e. lymphocytes).^38^ The concomitant changes observed in EC levels, *Collinsella* and cytokines suggest that this genus might exert some of its pro-inflammatory effects via modulating the EC system and it is possible that some of the effects of this genus on insulin resistance may also be linked to ECs given the tight relationship between EC modulation and insulin resistance.^39^

So far, no previous study examined the potential link between the EC system, exercise, and the gut microbiome. In a study with obese and normal weight women, high moderate to vigorous activity levels were not only higher in normal weight women but were also associated with higher OEA and AEA levels.^40^ Studies have shown that acute physical exercise increases circulating AEA, but not 2-AG, levels in humans.^41,42^ Two studies on elite rugby players showed that depending on diet and BMI the athletes had higher microbial diversity^43^ and increased fecal SCFA levels^44^ compared to non-active controls, whereas another study found cardiorespiratory fitness associated with higher levels of microbiome diversity, especially butyrate-producing bacteria independent of diet.^45^ While using small numbers of participants, a longitudinal study using 6 weeks of progressive endurance exercise without changing diet in overweight women found an increase in SCFA-producing bacteria,^46^ independent of age, weight, fat % as well as energy and fiber intake.^47^ Therefore, our data confirm findings from previous studies that have shown that ECs and SCFAs increase with exercise and further reveal the strong correlation between increases in ECs and decreases in pro-inflammatory cytokines. It is possible therefore that improved EC tone induced by exercise may be mediating the shift in the gut microbiota to increased SCFA producers, thereby increasing the SCFA production without a dietary change. This is a hypothesis that needs testing in a controlled experimental setting.

Using formal mediation analysis, we find that ECs are statistically mediating up to a third of the effect of SCFAs on the circulating levels of pro and anti- inflammatory cytokines. We further show that EC levels, specifically AEA and OEA, are positively correlated not just with EC system genes such as cannabinoid receptor 2 (CNR2) but also with higher expression levels of the SCFA receptors *FFAR2* but not with long fatty acid receptor like *FFAR4*. These data complement results from animal model studies showing that ECs can attenuate central and peripheral inflammation, can modulate gut microbiota composition and can reduce markers of gut permeability.^48^ CNR2 deficient mice had higher serum levels of the anti-inflammatory cytokine IL-10 compared to controls following a bacterial lipopolysaccharide challenge.^49^ The anti-inflammatory role of CNR2 was further demonstrated both in a chronic murine animal model and in IBD patients.^50^ FFAR2 was found to be necessary for the inulin-induced reduction of food-intake and protection against diet-induced obesity.^51^ Overall, our data confirm that ECs and SCFAs are crucial modulators of the effects of the gut microbiome on human metabolism and physiology.

Consistent with previous studies,^52–54^ we find anti-inflammatory effects of endocannabinoids, including positive associations of 2AG and OEA with anti-inflammatory markers (IL-10) and negative associations of most ECs with pro-inflammatory cytokines (TNFa and IL-6). At the same time, we show that the anti-inflammatory effects of ECs are partly mediated by increased levels of SCFAs and specifically butyrate. Elevated levels of butyrate have been shown to decrease mucosal permeability by increasing the secretion of mucins.^55,56^ Furthermore, ECs have emerged as important players in modulating gut permeability via enhancing the production of the tight-junction protein, occludin-1, as well as decreasing the expression of claudin-1, that serves as a paracellular barrier.^57,58^ Although further *in-vitro* and *in-vivo* studies are required to unravel the specific pathways involved, we think that the EC system and the gut microbiome play a role jointly in regulating an inflammatory status.

Our study has a number of strengths. Firstly, the findings from our study reinforce previous knowledge of an interaction between the EC system and the microbiome and add to previous literature by revealing an interaction between ECs, SCFAs and inflammatory system markers. Secondly, we have validated the cross-sectional associations between ECs and bacterial abundances in an independent cohort, and we have used gene expression data to annotate the pathways involved. Lastly, we have assessed the relationship between ECs, SCFAs and cytokines both cross-sectionally and longitudinally and have shown that simple lifestyle interventions such as exercise can modulate inflammatory markers via SCFAs and ECs.

We also note some study limitations. The transcriptomic assay we used did not include probes for *FFAR1* and *FFAR3* nor did it include *CNR1* making our gene expression data only partially informative. The exercise intervention we carried out was performed in individuals with pain in knee osteoarthritis and may not be directly relevant to other groups. However, the associations between ECs, SCFAs and cytokines were validated in a healthy age and sex matched smaller cohort suggesting that our data are generalizable. The findings presented from the formal mediation analysis are purely statistical and do not indicate causality.

In conclusion, in this study we show that circulating levels of ECs are consistently associated with higher levels of SCFAs, with higher microbiome diversity and with lower levels of the pro-inflammatory genus *Collinsella*. We also show statistically that the anti-inflammatory effects of SCFAs are up to one third mediated by the EC system.

## Supplementary Material

Supplemental Material

## Data Availability

The data that support the findings of this study are available at www.ebi.ac.uk/metabolights/MTBLS3203.
