# Supplementary material for: The anti-inflammatory effect of bacterial short chain fatty acids is partially mediated by endocannabinoids
Source: Gut Microbes. 2021 Nov 17;13(1):1997559. doi: 10.1080/19490976.2021.1997559 (PMC8604388; doi:10.1080/19490976.2021.1997559)
Supplement: Supplemental Material [file KGMI_A_1997559_SM6117.zip › Supplementary information/Supplementary figure captions.docx]

**Supplementary files**

**Supplementary Figure 1:** Matrix plot showing the associations of: (A) OTU abundance and short chain fatty acids with endocannabinoids; and (B) pro and anti-inflammatory markers with endocannabinoids in the cross-sectional independent cohort. Squares represent beta coefficients with size and colour varying based on size and direction of association. (FDR adjusted *p<0.05)

**Supplementary Figure 2**: Heat map plot of positive (red) and negative (blue) associations of change in ECs with change in SCFAs. None of the associations were statistically significant after FDR correction (p > 0.05).
