# Supplementary figures and images for: The anti-inflammatory effect of bacterial short chain fatty acids is partially mediated by endocannabinoids
Source: Gut Microbes. 2021 Nov 17;13(1):1997559. doi: 10.1080/19490976.2021.1997559 (PMC8604388; doi:10.1080/19490976.2021.1997559)

## Slide 1
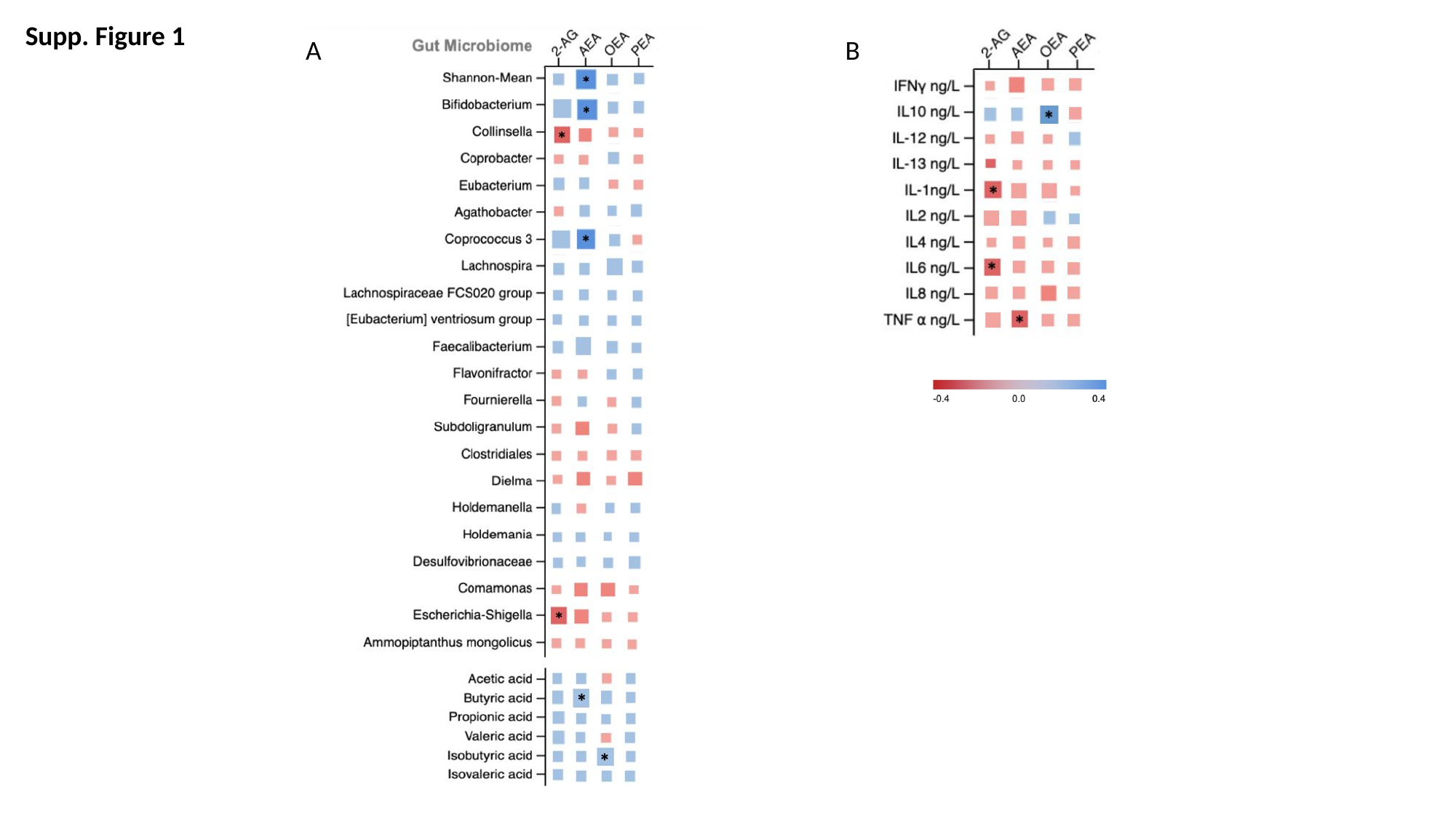

Supp. Figure 1
A
B

## Slide 2
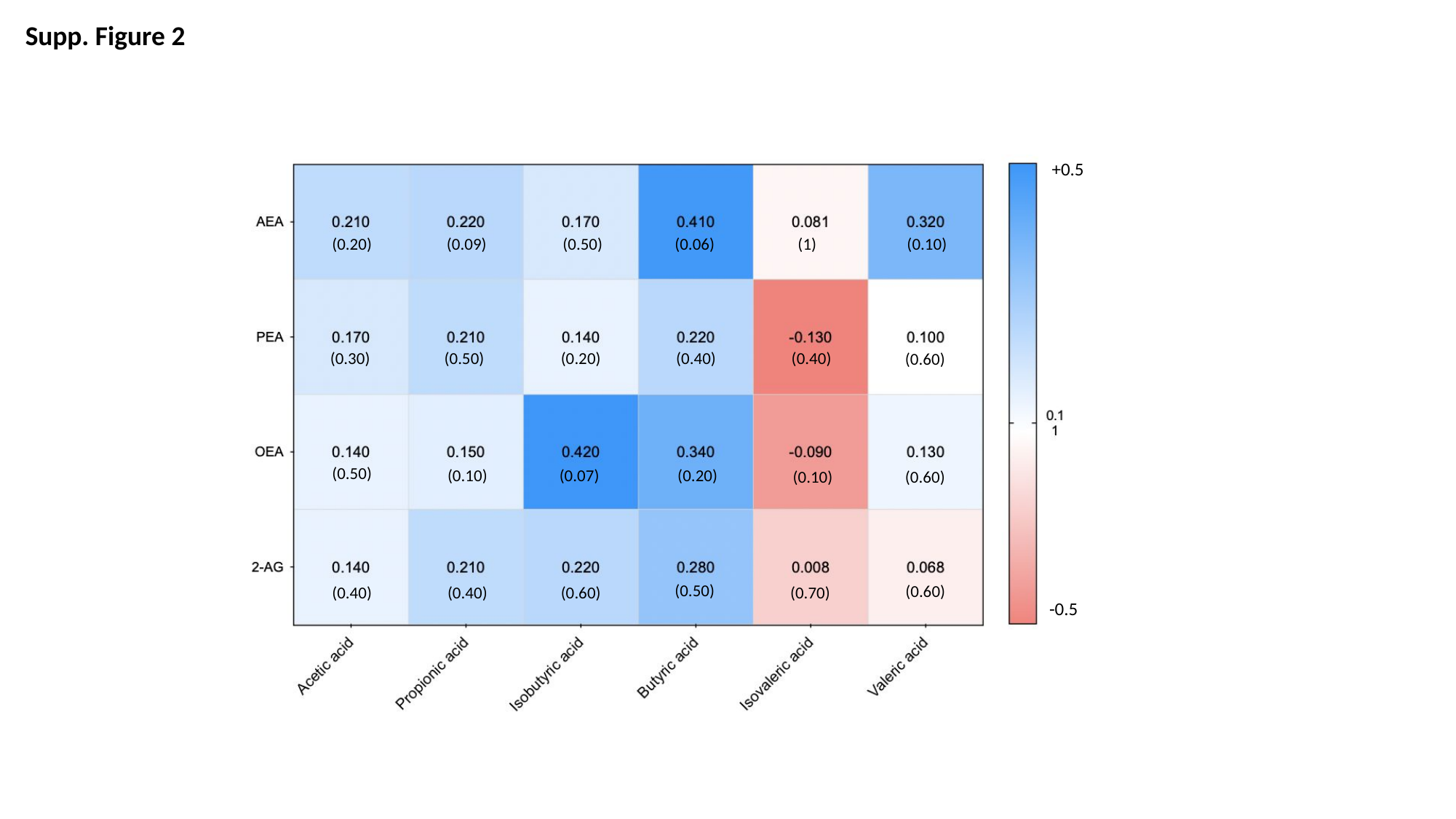

Supp. Figure 2
(0.20)
(0.09)
(0.50)
(0.06)
(1)
(0.10)
(0.30)
(0.50)
(0.20)
(0.40)
(0.40)
(0.60)
(0.50)
(0.10)
(0.20)
(0.07)
(0.10)
(0.60)
(0.50)
(0.60)
(0.40)
(0.40)
(0.70)
(0.60)
+0.5
-0.5

Supplement: Supplemental Material [file KGMI_A_1997559_SM6117.zip › Supplementary information/Supplementary figures_EC_revised.pptx]
